# Sequential IgE-epitopes of ovomucoid exposed by glutathione are relevant in persistent egg allergy

**DOI:** 10.1186/2045-7022-3-S3-P173

**Published:** 2013-07-25

**Authors:** T Kinaciyan, F Roth-Walter, P Starkl, T Zuberbier, R Brunner, IP-S Pali-Schöll, J Kinkel, FF Felix, E Jensen-Jarolim

**Affiliations:** 1 DIAID, Dept of Dermatology, Medical University of Vienna, Vienna, Austria; 2 Messerli Forschungsinstitut, Veterinärmedizinischen Universität Wien, Vienna, Austria; 3 Institute of Pathophysiology and Allergy Research, Medical University of Vienna, Vienna, Austria; 4 Department of Dermatology, Charité Universitätsmedizin, Berlin, Germany

## Background

The egg allergen ovomucoid (Gal d 1) is conformationally stabilized by 9 disulfide bonds. However, patients with persistent egg-allergy have more IgE against sequential than conformational epitopes of ovomucoid. We investigated whether natural or added food compounds may cause reduction of disulfide bonds and linearization of ovomucoid.

## Methods

Reduced ovomucoid in raw eggwhite was detected using a fluorescence-labeled alkylation probe. The common anti-oxidants glutathione and cystein were used for in vitro linearization and effects were monitored by CD-spectrometry. Egg-allergic patients were tested serologically (n=19) and by skin prick test (n=9) for IgE against native and linearized ovomucoid and optionally its cooked state.

## Results

Linearized ovomucoid could be detected in native eggwhite. Glutathione and cystein treatments, but not cooking linearized ovomucoid, as confirmed by CD-spectrometry. In Western Blot involving cooking more patients had IgE against reduced than native ovomucoid. In ELISA, most IgE was found against raw and native ovomucoid. Cooking of native ovomucoid significantly decreased, whereas cooking of previously linearized ovomucoid enhanced IgE-binding. In skin prick test 5/9 patients reacted with linearized ovomucoid.

## Conclusion

Linearized ovomucoid is present in natural eggwhite. Glutathione which is occurring naturally but is also frequently used as structure improving additive in processed food is partly responsible. Additional cooking of linearized ovomucoid increases IgE-reactivity in patients with persistent egg-allergy in vitro and in skin prick tests. Our data provide evidence that reduction is a novel principle which contributes to the allergenicity of food. This may be relevant for new allergies to modern processed food.

## Disclosure of interest

None declared.

